# The Static Limit in MeV Secondary Ion Mass Spectrometry

**DOI:** 10.1021/jasms.5c00024

**Published:** 2025-03-07

**Authors:** Mirjana Sepahyar Lorentzen, Boštjan Jenčič, Primož Vavpetič, Primož Pelicon

**Affiliations:** † Jožef Stefan Institute, Jamova 39, 1000 Ljubljana, Slovenia; ‡ Jožef Stefan Institute Postgraduate School, Jamova 39, 1000 Ljubljana, Slovenia

## Abstract

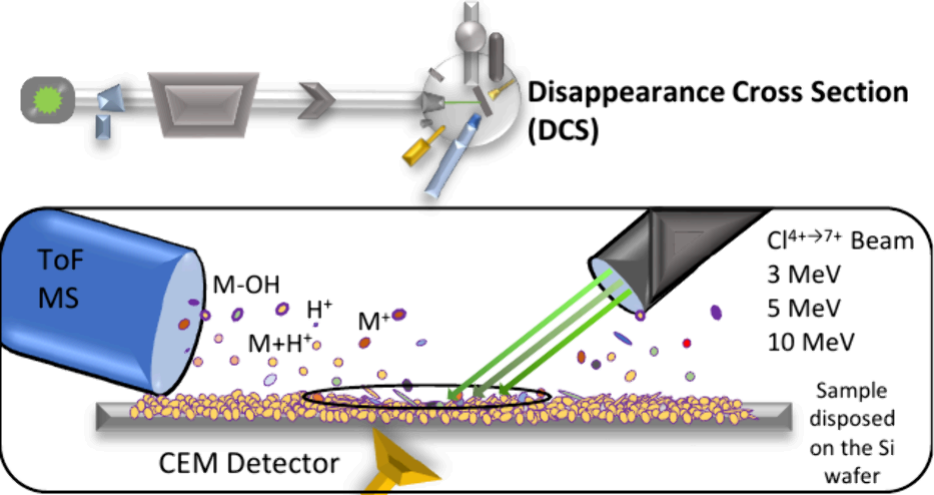

Static limit in secondary ion mass spectrometry (SIMS) is defined as a threshold beam fluence, where secondary ions are desorbed only from the virgin surface. For the common SIMS technique, the static SIMS limit is set to approximately 10^12^ ions/cm^2^. Within the present paper, we investigated the applicability of the static limit for a mass spectrometry imaging technique known as MeV-SIMS, where the target surface is bombarded by primary ions within the MeV energy range domain. Here, desorption of secondary ions relies mainly on electronic excitations instead of collision cascades, as is the case for the lower energy primary ion beams. We have measured the disappearance cross sections of several organic targets for three different chlorine primary ion beam energies. Results show how the disappearance cross section depends on the primary ion beam energy. Generally, the static SIMS regime applies for a range of primary ion beam fluences similar to that for the common SIMS technique; however, the dependence of the disappearance cross section within the lower MeV energy domain (up to 10 MeV) exhibits somewhat unexpected characteristics. Further, we thoroughly investigated the dynamics of the secondary ion mass spectra after prolonged primary ion bombardment. Secondary ion yields of various peaks were monitored during analysis, and the corresponding data can be used to identify specific peaks and also to determine fragmentation patterns of analyzed organic molecules.

## Introduction

Secondary ion mass spectrometry (SIMS)^1,2^ is a routinely used mass spectrometry imaging (MSI) technique, which provides superior lateral resolution and is therefore a useful tool for molecular investigations of inorganic and organic samples on a submicron scale.^3^ It is based on interactions between the primary ion and the target material, which result in the emission of molecular electrons from the target surface. Such interactions cause chemical alteration of the surrounding area, and their reach defines the disappearance cross section (σ^2^) for a specific primary ion.

Static SIMS limit is defined as a beam fluence (number of primary ions per area unit), where damaged spots on the sample surface are few and far apart from each other, so each new primary ion is very likely to probe a chemically unaltered sample. For the classic SIMS, the static SIMS limit is set to approximately 10^12^ ions/cm^2^,^4^ where the ratio of damaged sample stays at approximately one percent.

Aiming for better secondary ion yields (SIY) of large, intact biomolecules, larger primary ion clusters, such as SF_5_, C_60_, Ar_*n*_, (H_2_O)_*n*_, and (CO_2_)_*n*_ were introduced with great success regarding SIY enhancement.^5−10^ Although the disappearance cross sections for such clusters were measured as slightly larger for SF_5_^11^ and C_60_,^9^ their efficiency (defined as a ratio between secondary ion yield and disappearance cross section) is still much higher than for monatomic primary ions. The improved efficiency specifically enhanced the detection success of larger organic molecules with masses up to 10 kDa.

Similarly, the use of faster, MeV, primary ions also results in higher secondary ion yields and lesser fragmentation of larger, organic molecules.^12,13^ The method is known as MeV-SIMS. Coupled with other high-energy focused ion beam analysis (IBA) techniques, such as particle induced X-ray emission (PIXE or micro-PIXE), this feature makes MeV-SIMS an appealing technique for research in biology, biochemistry, medicine, and also forensics, where complementary information about elemental and molecular distribution is required. Recent improvements in MeV-SIMS capabilities^14^ at Jožef Stefan Institute tandem accelerator facility made understanding of some fundamental processes even more desired, since the quest for multimodal imaging^15^ requires a thorough understanding of how each IBA method affects the sample.

Disappearance (damage) cross section measurements for fast heavy ions were already conducted by Eriksson et al.^16^ In their study, organic targets, such as valine, leucine, trileucine, insulin, and LHRH (lutenizing hormone releasing hormone) peptide, were bombarded by various primary ions with energies of approximately 70–80 MeV. Results exhibited a linear increase of disappearance cross section with primary ion stopping power (d*E*/d*x*), such as predicted by the hit theory for fast-ion-induced desorption of molecules.^17^ However, these energies are significantly higher than the energies of most facilities where MeV-SIMS and PIXE analyses are being conducted. Most MeV-SIMS setups rely on primary ion beams of energies up to 10 MeV. At such lower energies, electronic stopping is already the dominant sputtering process, yet nuclear stopping (collision cascades) is not completely negligible and was shown to significantly influence fragmentation of larger organic molecules.^13^ Therefore, a more detailed understanding of the dependence of disappearance cross section on the primary ion energy and the corresponding stopping power is required in order to fully exploit the advantages of MeV-SIMS.

## Methods

### 1 Sample Preparation

All samples were purchased from Sigma-Aldrich and were separately, each analyte by itself, prepared as water solutions, and later deposited (ca. 1 μL) and spin-coated (2000 rpm for 30 s) on a polished silicon wafer. Based on previous experiments, such parameters provide a sample with a thickness of more than 100 nm, which is thick enough to prevent interference of silicon during measurements.

Arginine (MW = 174 Da) was dissolved at 5 g/100 mL, leucine (MW = 131 Da) at 3 g/100 mL, leu-enkephalin (MW = 555 Da) at 3 g/100 mL, and cholesterol (*m* = 386 Da) at 1 g/100 mL. Polyethylene glycol (PEG) 600 was mixed with lithium trifluoroacetate (LiTFA), and PEG 1000 was mixed with silver trifluoroacetate (AgTFA), both at 1:1 m/m ratio, in order to enhance the secondary ion yield. Such samples were dissolved in water at 5 g/100 mL.

The size of silicon wafers was approximately 1 cm × 1 cm, which allowed multiple analysis on the same sample. Spots for the analysis were distanced at least 1 mm from each other.

### 2 MeV-SIMS Analysis

All measurements proceeded by employing the 2 MV tandem accelerator at the microanalytical center of Joef Stefan Institute. Cl^–^ ions of 30 kV were generated in the sputter ion source and afterward accelerated in the tandem accelerator to energies between 3.2 and 10.0 MeV in the form of positively charged ions. Corresponding charge states of ions with the aforementioned energies ranged between 4+ and 7+.

A primary ion beam was pulsed with a frequency of 10 kHz, and the duration of pulses was approximately 10 ns. It was focused to dimensions of approximately 15 × 15 μm^2^, first by closing the object and collimator slits (mechanical apertures) and finally by utilizing a triplet of quadrupole magnetic lenses. The focused beam bombarded the target at an angle of 55°. The voltage on the target was +3 kV; therefore only positive secondary ions were accelerated in a linear time-of-flight mass spectrometer, with a microchannel plate (MCP) detector at its end. The mass resolving power of the acquired mass spectra was estimated to m/dm = 600 at *m*/*z* = 174 (arginine peak).

Primary ion beam intensity was monitored with a channel electron multiplier (CEM) detector positioned at the axis of the beam. In the pulsed mode, primary ion intensity was between 2000 and 15 000 Hz, which translates to the range between 2 and 10 fA (considering also the variable charge state of the beam). The total ion dose was then estimated through the measurement of the primary ion beam intensity before and after the analysis. The current was found to be stable over a period of 10 h, and the variations were less than 6%. We also continuously monitored the beam current from the sputter ion source over the entire duration of the measurement, which guaranteed that there were no sudden spikes in the ion beam intensity.

### 3 Disappearance Cross Section Measurements

Disappearance cross section was measured for the aforementioned organic samples, which were bombarded by primary ion beams of three different energies and their corresponding charge states: 3.2 MeV Cl^4+^, 5.0 MeV Cl^5+^, and 10.0 MeV Cl^7+^. All three types of beams were focused to a similar size: 17 μm × 15 μm (3.2 MeV), 14 μm × 19 μm (5.0 MeV), and 14 μm × 16 μm (10.0 MeV). The profiles of the beam were estimated by scanning the negative image of the copper mesh with 250 periods per inch.

Beam intensity was decreasing with increased energy due to the lesser probability of acquiring a higher charge state. A 3.2 MeV beam had an intensity of 8.0–12 kHz, 5.0 MeV 4.0–6.0 kHz, and 10.0 MeV 2.0–3.0 kHz. The intensity was monitored before and after each measurement and was found relatively stable (1 ± 0.06) for a prolonged time.

Each measurement was done on a single spot (equal to the size of the beam) in order to make the acquisition quicker, and their duration was such that approximately 10^8^ ions bombarded the spot, which lasted several hours. Although this yields the average fluence between 10^13^ and 10^14^ ions/cm^2^, the fluence was not homogeneous across the bombarded spot due to the primary ion beam having a two-dimensional Gaussian distribution in intensity. The overall yield over time (or number of bombarded ions) can be estimated as
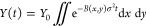
1where *Y*_0_ is the initial secondary ion yield, σ^2^ is the damage cross section, and
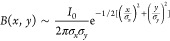
2Here, *I*_0_ is the ion count rate and σ_*x*,*y*_ is the parameter of the Gaussian curve size. The actual dimensions of the beam are approximately 2.14 × σ_*x*,*y*_ and were determined as a distance between points, where the beam intensity falls to 10% of its maximum intensity.

Borders of the two-dimensional integral are determined by collimator slit apertures, which are opened by approximately 0.3 mm in each direction.

## Results and Discussion

**Figure 1:**
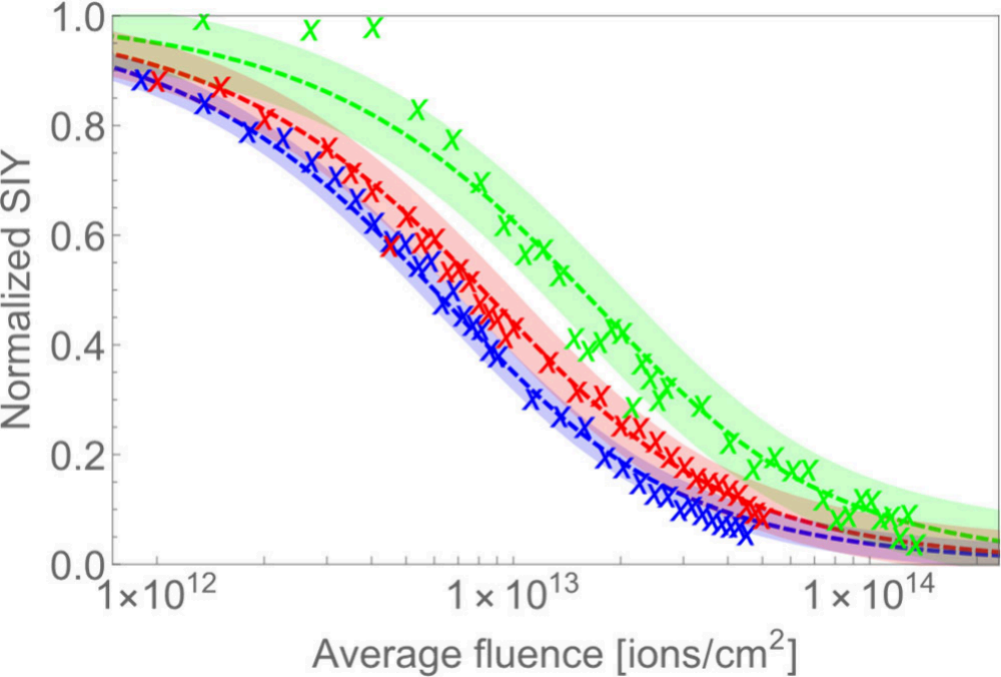
Secondary ion yields as a function of average beam fluence for the protonated leucine enkephalin molecular peak (*m*/*z* = 556). Three curves represent dependence of SIY for three primary ion beam energies: 3.2 MeV (blue), 5.0 MeV (red), and 10.0 MeV (green). The fitted curves correspond to σ^2^ = 5.8 nm^2^ (blue), 4.4 nm^2^ (red), and 2.0 nm^2^ (green). The SIY curves are normalized to the initial secondary ion yield for each of the three measurements.

The correspondence of experimental results and theoretical prediction of eq 1 is shown in Figure 1, which depicts the secondary ion yield as a function of average beam fluence for protonated leucine-enkephalin (*m*/*z* = 556). Similar curves for other compounds can be found in the Supporting Information. Figure 1 demonstrates how the secondary ion yield decreases at different rates when bombarded by ion beams of different energies. All data are normalized to *Y*_0_ (initial secondary ion yield) for a given energy of the primary ion beam. We defined average beam fluence as a number of all ions per area unit, where area unit was estimated by knowing the size of the beam, in order to easily assess the data. However, it is important to note that the beam fluence is not spatially homogeneous, and the centermost region was exposed to much higher doses than the borders of the analyzed spot.

Different rates of secondary ion yield decrease correspond to σ^2^ = 5.8 ± 0.5 nm^2^, 4.4 ± 0.5 nm^2^, and 2.0 ± 0.4 nm^2^, with the highest damage cross section being observed for the lowest primary ion energy of 3.2 MeV (91.4 keV per nucleus), presented in blue. A significant part of the error is associated with variation of the ion beam intensity measurements.

**Figure 2:**
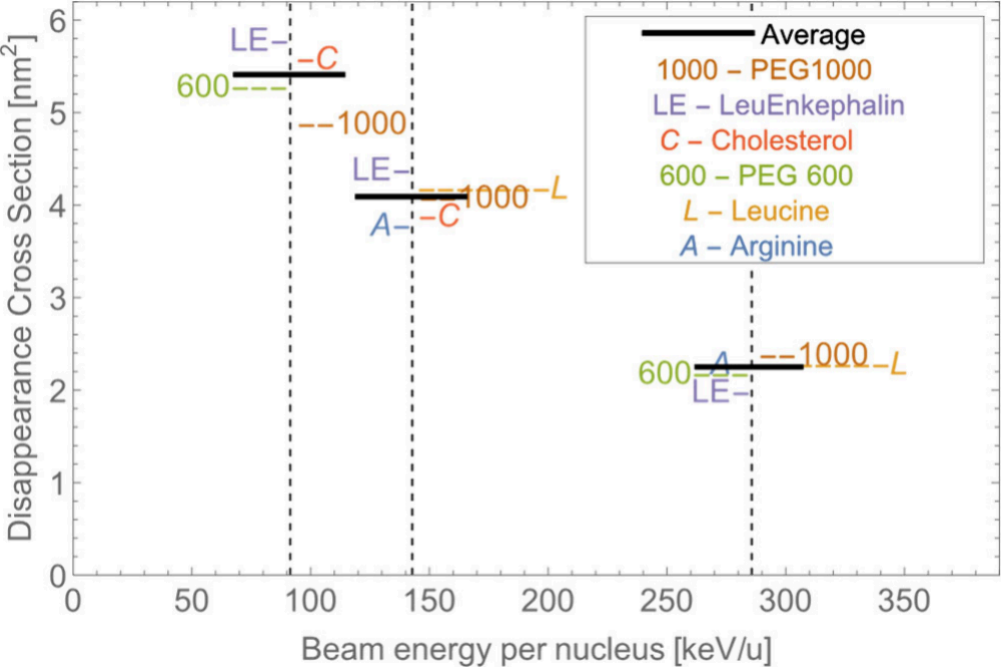
Measured disappearance cross sections for various organic molecules at different primary ion energies. The average σ^2^ is 5.4 nm^2^ for 3.2 MeV (91.4 keV per nucleus), 4.1 for 5.0 MeV (142.9 keV/u), and 2.2 for 10.0 MeV (285.7 keV/u).

Disappearance cross sections on other reference samples, such as arginine, PEG, leucine, and cholesterol, were also measured with values similar to that for leucine-enkephalin and are presented in Figure 2. Similarly, the decrease of the disappearance cross section with increasing primary ion energy can be observed, and such behavior seems universal for all organic molecules within the analyzed energy range. The average σ^2^ for the 3.2 MeV primary ion beam is 5.4 ± 0.5 nm^2^, while the average σ^2^ values decrease to 4.1 ± 0.5 nm^2^ for 5.0 MeV energy and 2.2 ± 0.4 nm^2^ for the energy of 10.0 MeV.

This result is somewhat unexpected, since the impact of the fast-heavy ion should increase proportionally to the square root of energy per nucleus of the primary ion beam.^18,19^ Such a dependence should apply for both types of regions, which are differently affected by the primary ion.

The smaller region, known as the “infra-track”, is where the beam interferes directly with the target and where high-energy electrons are produced and later carry the excitation further out in the material. The damage to the material within this area is substantial for any beam energy. However, this region is quite small, since its radius was empirically estimated as 0.67(*E*/*M*)^1/2^ nm, where *E* is the beam energy in MeV and *M* is the mass in atomic units. This translates to an area between 0.1 and 0.4 nm^2^ for 0.1 and 0.3 MeV/u (3 to 10 MeV ^35^Cl), much smaller than measured values of σ^2^.

Large differences between measured disappearance cross sections and the expected area of the infra-track suggest that the most significant contributor to the effective disappearance cross section is the larger region, known as the “ultra-track”. This area is where secondary ions emerge from and is only indirectly affected by the primary ion. For given beam parameters, the area of the ultra-track ranges between 10 and 100 nm^2^; therefore we speculate that the obtained values of σ^2^ are severely affected by the rate of fragmentation within the ultra-track, which is also dependent on the primary ion energy.

Measured values confirm the validity of the commonly accepted “static-SIMS” limit at 10^12^ ions/cm^2^ also for MeV-SIMS. Under the assumption of a static regime, where each spot is only bombarded once (damage remains small), 10^12^ ions/cm^2^ result in the damaged area of 10^12^ × σ^2^ over 1 cm^2^, which ranges between 0.022 cm^2^ for a 10 MeV beam and 0.054 cm^2^ for a 10 MeV beam, or between 2% and 5% of the surface area, which is similar to the damage induced by the common (keV) SIMS technique.

The data, presented in Figure 2, show no correlation between σ^2^ and the size of the molecule. This can be additionally verified by observing σ^2^ of several molecular peaks within the PEG spectra, where, for the same sample, the curves of SIY as a function of the primary ion dose are very similar.

During the prolonged bombardment, ratios between specific peaks also change significantly, with some peaks decreasing in intensity and others, mostly representing fragments, increasing even after very high beam fluences. We performed a detailed analysis of various peaks within the mass spectra of the PEG 1000/AgTFA 1:1 sample, bombarded by a 5 MeV primary ion beam, where several molecular peaks (labeled *M*_n_) are present along with signals of fragments of various sizes. Our goal was to evaluate each peak in accordance with its yield dependence from the primary ion beam fluence.

**Figure 3:**
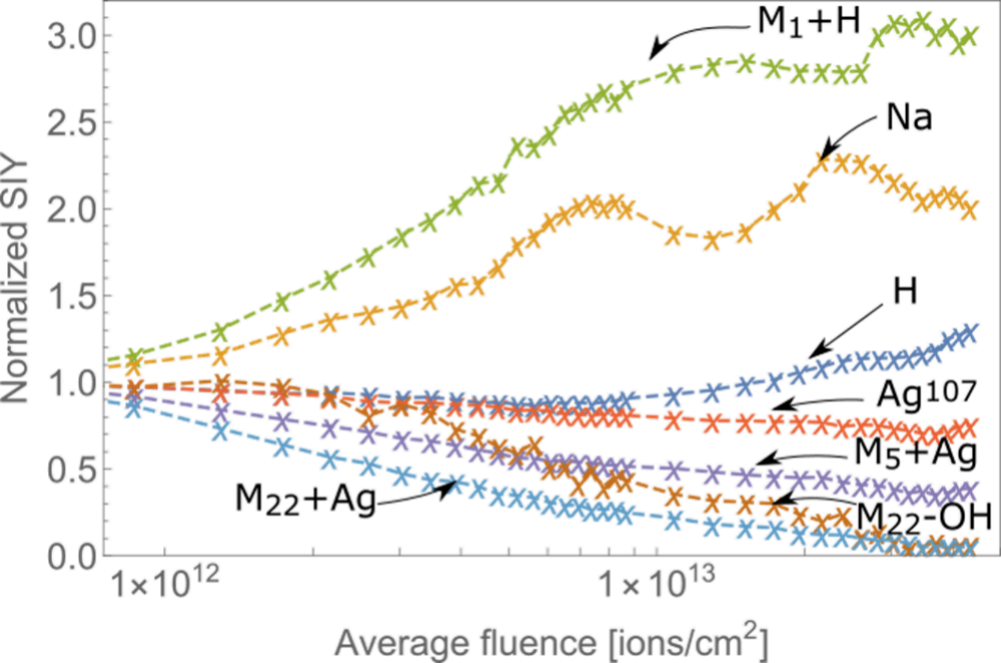
Normalized secondary ion yield of various peaks within PEG 1000/AgTFA spectra, measured by a 5 MeV Cl^5+^ ion beam, as a function of average beam fluence.

Typically, molecular peaks exponentially decrease in intensity, as illustrated in Figure 1 for the case of leucine-enkephalin. However, Figure 3 shows that only molecular peaks act in accordance with eq 1, while the SIY of other peaks has a more unpredictable dynamics, which can give additional information about their origin.

Figure 3 shows peaks of various elements: hydrogen, sodium, and the ^107^Ag isotope. The signal of these peaks remains relatively stable across the entire bombardment procedure, although sodium exhibited a more significant increase. Most likely, the change of these secondary ion yields is due to different ratios between ejection of positive, neutral, and cluster forms of elements. Such an occurrence can be most notable with elements that are detected in their ionized state or in a form of various clusters, contrary to secondary molecules, where a vast majority are detected in a protonated or cationized state, and cluster formation is not as likely.

Especially sodium was found to exhibit a significant increase of the secondary ion yield after the long exposure, which was also the case for some related ionic clusters, such as Na_2_Cl, Na_3_Cl_2_, and Na_2_OH. The same pattern also appeared in all potassium-based peaks. We speculate that this increase can be attributed to charging of the sample, which might have catalyzed the production of secondary ions from the alkali metal group.

A typical example of the yield increase is that of the M_1_+H peak, which represents a protonated base block (C_2_H_4_O) of the polyethylene glycol molecule. It is therefore the most common fragment of a PEG molecule, although not necessarily does a larger (around the size of *m*/*z* = 1000) molecule decay to a base block.

Medium-sized fragments and intact PEG 1000 molecules (M_*n*_+H_2_O, *n* = 16–28) exhibit a decrease in the secondary ion yield; however, the decrease comes at different rates. An example of a medium-sized fragment is M_5_+Ag (*m*/*z* = 345), which has a significantly lower decrease than that of larger intact molecules. Still, the similarity between a curve of this fragment is still very high in relation with parent peaks. On the other hand, a second set of PEG peaks, M_*n*_–OH, has a distinctive dependence on the beam fluence, since it does not start decreasing until the average beam fluence exceeds 2 × 10^12^ ions/cm^2^, but later decreases even faster than a set of M_*n*_+Ag peaks.

The secondary ion yield dependencies for different peaks, as seen in Figure 3, were found very similar when bombarding separate PEG 1000/AgTFA 1:1 samples with primary ions of different energies and were also similar for other organic molecules, such as leu-enkephalin.

An origin of the individual peak within the mass spectrum can be traced through comparing the correlation of its SIY dependence on the average ion dose in relation to one of the parent molecule peaks.

The correlation between the two functions was calculated as
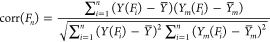
3where *F*_*i*_ is the cumulative average fluence at the *i*th consecutive measurement of the secondary ion yield, *F*_*n*_ is the final beam fluence of the analysis, *Y* is the secondary ion yield of the analyzed peak, and *Y*_*m*_ is the secondary ion yield of the molecular peak M_21_+Ag. *Y̅* and *Y̅*_*m*_ are arithmetic averages of functions *Y*(*F*_*i*_) and *Y*_*m*_ (*F*_*i*_) up to *F*_*n*_.

**Figure 4:**
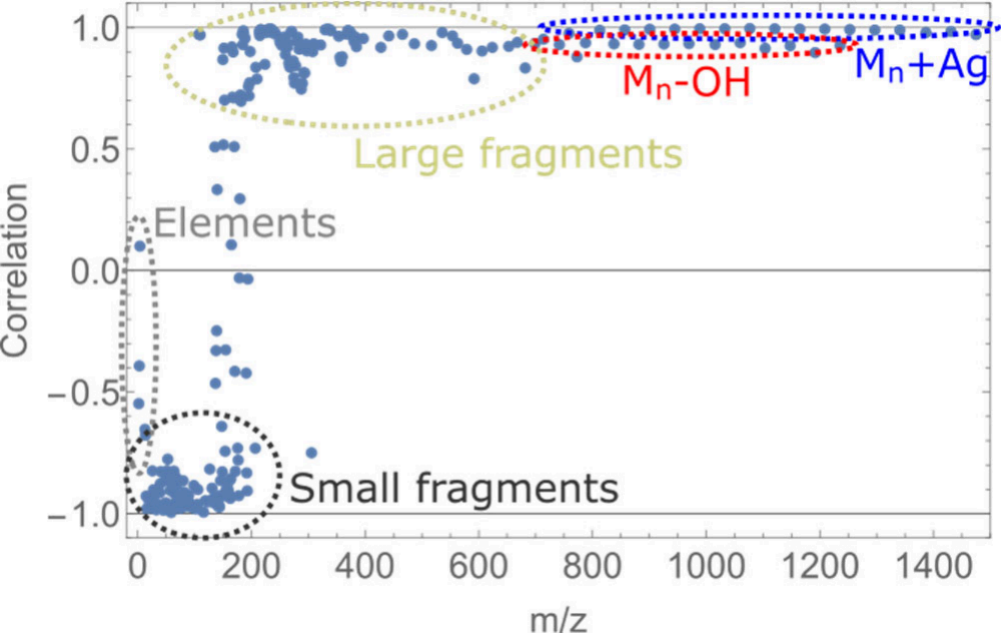
Correlation plot for peaks within the PEG 1000/AgTFA mass spectra after a long exposure (avg. dose > 10^14^ ions/cm^2^). Reference is M_21_+Ag. A correlation close to +1 means similar behavior of the secondary ion yield as a function of time in relation to the M_21_+Ag molecular peak. A correlation closer to −1 means an increase of secondary ion yield over time. A clear separation between M_*n*_+Ag and M_*n*_–OH peaks can be observed, as well as a separation between large, medium-sized, and small fragments. Elements, such as H, Na, and Ag, are mostly not strongly correlated to the molecular peak.

The value of corr(*F*_*n*_) therefore depends on *F*_*n*_ and should converge to +1 for all peaks in the spectrum when *F*_*n*_ → ∞. We have measured the secondary ion yield dependency up to average beam fluences of approximately 10^14^ primary ions/cm^2^, where, as can be seen in Figures 1 and 3, the intensity of molecular peaks was significantly reduced after a steady exponential decrease. However, *Y*(*F*) curves for other peaks were still not closely correlated to *Y*_*m*_(*F*). Correlations for peaks within the PEG 1000/AgTFA spectra after the highest achieved fluence are listed in Figure 4.

In Figure 4, the distinctive groups of peaks can be visible more clearly when separated through their correlation with the molecular peak. All M_*n*_+Ag peaks are closely correlated (corr > 0.99) with each other, while M_*n*_–OH peaks all have a correlation of approximately 0.92 with intact molecular peaks. It is apparent from Figure 3 that functions *Y*(*F*) for M_*n*_–OH peaks have a delayed onset of decrease, which causes a somewhat smaller correlation.

Afterward, larger fragments vary more with their correlation, since some are increasing at the beginning and afterward start to decrease, while others are decreasing from the beginning and are therefore better correlated to parent peaks. Nevertheless, they all have a correlation of greater than zero, which means their intensity already starts to significantly decrease after the irradiation with 10^14^ primary ions/cm^2^. On the other hand, small fragments have a distinctively negative correlation, meaning they are increasingly produced even after a longer bombardment of the sample.

**Figure 5:**
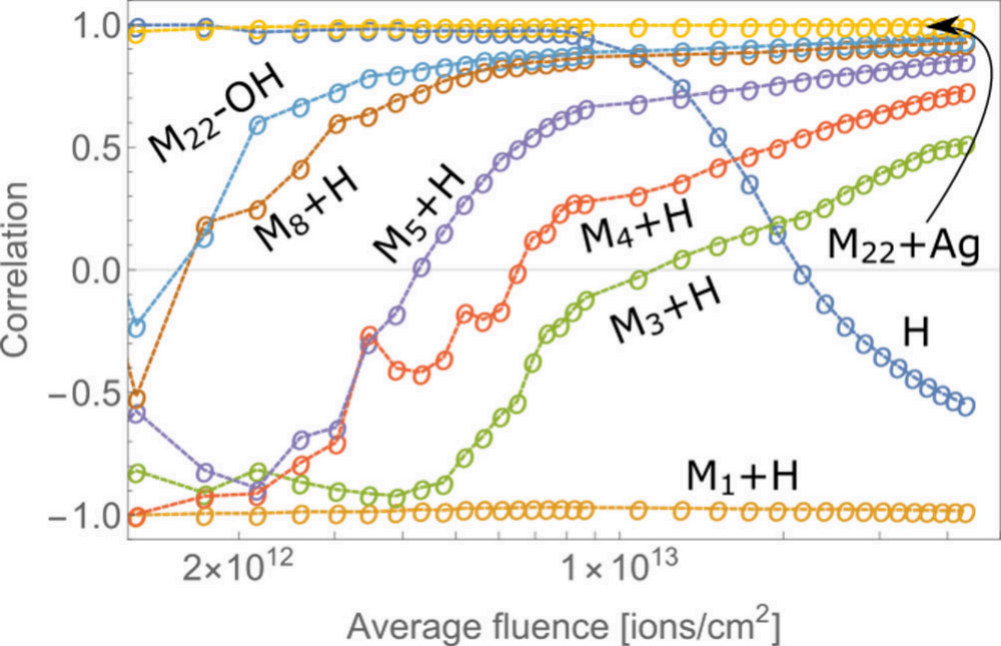
Correlation of *Y*(*F*) curves as a function of the average beam fluence. Reference curve is that of M_21_+Ag. All other molecular peaks (M_*n*_+Ag, *n* = 16–28) are well correlated at all times, while smaller fragments require a specific dose in order for their correlation to become positive. The required dose increases as the size of the fragment decreases.

As described in eq 3, the correlation between curves also changes over time with increasing the beam fluence, and the intensity of most peaks (molecular or fragment) is expected to converge to zero in the high fluence limit. However, the rate of reaching the limit is different from molecule to molecule and highly mass dependent, as illustrated in Figure 5.

Here a correlation between SIY curves of several peaks and the M_21_+Ag peak is depicted as a function of the average beam fluence. For the case of M_22_+Ag, the correlation is very close to 1 during the whole bombardment process, as is expected, since the curves are similar at any time and both peaks represent the original molecule. Similarly correlated are also secondary ion yields from all other molecular peaks, which again confirms that the size of a molecule does not directly affect the disappearance cross section. Further, an *F*(*Y*) of similarly sized molecule M_22_–OH was initially even negatively correlated to the *F*(*Y*) of molecular peaks, hinting at a minor production of such fragments with low primary ion doses. However, this production soon becomes irrelevant, since such molecules are very likely to fragment more than just losing an OH group, and later the correlation converges to 1.

For smaller fragments, the pattern is similar; however, as the size of the molecule decreases, it takes increasingly more time (fluence) for the correlation to become positive. For M_8_+H (*m*/*z* = 371) the necessary average fluence is approximately 1.7 × 10^12^ ions/cm^2^, for M_5_+H (*m*/*z* = 239) it is 4.3 × 10^12^ ions/cm^2^, then 6.5 × 10^12^ ions/cm^2^ for M_4_+H (*m*/*z* = 195), and 1.2 × 10^13^ ions/cm^2^ for M_3_+H (*m*/*z* = 151). For the smallest fragment of M_1_+H, the correlation is strictly negative and may stay this way for very large doses that are at least a magnitude higher. Under the fluence of 10^14^ ions/cm^2^ each spot should be on average affected by the primary ions 2–5 times, which is presumably still not enough to break down the original molecule to just base blocks. A sample related element, such as H, has, on the contrary, at first a positive correlation, which stays close to 1 for doses up to 3 × 10^7^ ions, but afterward the correlation becomes negative.

## Conclusion

The results demonstrate that both the commonly used keV SIMS and MeV-SIMS can operate within similar boundaries regarding the primary ion beam fluence, since measured disappearance cross sections within this work also suggest a static limit at approximately 10^12^ ions/cm^2^. However, due to fundamental differences, the two techniques are appropriate for different applications. Since fast-heavy ions produce damage much beyond the mere surface of the sample, a 3D analysis, e.g., through sputtering several layers with an Ar beam, is not applicable or can only be efficient if the individual layer receives a dose of 10^11^ primary ions/cm^2^ or less. On the other hand, efficiency of MeV-SIMS is significantly higher due to operating with a very similar disappearance cross section but also gaining a secondary ion yield of approximately 10^–2^. For the static SIMS limit, one expects to obtain approximately 10^4^ ions/μm^2^, and 1 × 1 μm^2^ is the typical pixel size for tissue analysis. Therefore, sufficient statistics within the static-SIMS regime can only be achieved with an SIY above 10^–3^ secondary ions/primary ion, a feature that can only be achieved by MeV-SIMS or gas cluster ion beam SIMS, such as large argon, water, or CO_2_ clusters. So, although a static-SIMS limit is very similar for all types of primary ions and their energies, the number of desorbed secondary ions per area unit can differ greatly and is of higher importance.

The disappearance cross section was found to be dependent on the primary beam energy, as it is higher with lower energies. This result is not in line with the previously published work, where the absolute size of primary ion induced tracks in the sample was found to increase with increased energy of the ion. However, the disappearance cross section is related not only to the radius of the track but also to the produced damage within the track. One can expect that the smaller region, the so-called infra-track, is left with complete fragmentation of material within. However, the radius of this region is quite small and almost negligible compared to measured areas of damage. Therefore, the more important contribution is a larger region known as ultra-track, from where the secondary ions emerge. The area of the ultra-track is much larger than the effective damage cross section; therefore the rate of fragmentation is of great importance and can severely influence σ^2^. While some secondary ions might be desorbed and detected as either intact molecules or fragments, some material might also be damaged and desorbed only later with another incoming primary ion, which might result in observing larger σ^2^ for beam parameters, where this happens more frequently. Since the rate of fragmentation was found to decrease with increasing primary ion energy, such a decrease of σ^2^ could be explained by this.

An additional parameter that might influence the results is the charge state of the primary ion, which was higher for higher energies. However, for the same setup and chlorine ion species, the charge state was found not to influence the secondary ion yield and the fragmentation rate in organic molecules.^13^ Since both secondary ion yield and fragmentation rate are somewhat correlated to the disappearance cross section, we speculate that it is unlikely for the charge state to have a significant impact on results. Further studies regarding this might still be of interest. Within the work, we also demonstrated how the results of prolonged exposure to the primary ion beam can assist in interpretation of the spectra and the fragmentation processes. Different rates of secondary ion yield decrease or increase are highly dependent on the fragmentation pattern of the original molecule. For the case of PEG 1000, all molecular peaks M_*n*_+Ag exhibited the same decrease of secondary ion yield; however, even the curves for M_*n*_–OH fragments demonstrated some offset, which suggests that some fragments might be produced from the first interaction of the primary ion beam with the target and are desorbed only later, when their proximity is bombarded for the second or even third time.
